# Supplementary material for: Hypotony after 25-gauge pars plana vitrectomy with suturing all sclerotomies
Source: PLoS One. 2026 Feb 11;21(2):e0341951. doi: 10.1371/journal.pone.0341951 (PMC12893548; doi:10.1371/journal.pone.0341951)
Supplement: Attachment — Submitted filename: Response to Reviewers.docx [file pone.0341951.s003.docx]

Editor Comment:

Significant issues have been raised regarding the absence of a study control group in which sutures were not used, therefore preventing acceptance of the concluding claim that sutured sclerotomies reduce hypotony.

Answer:

Thank you for your thoughtful observation. To avoid giving the impression of being overstated, we revised the wording from ‘reduced hypotony’ to ‘had a low incidence of hypotony’ and to ‘had no incidence of irreversible visual impairment due to severe hypotony’. These revisions were made in the Discussion section (Lines 165 and 220).

Also, there are concerns about the study design, which assessed hypotony only on the first postoperative day and lacked longitudinal follow-up. This eliminates some patients with a clinical subset of hypotony.

Answer:

We included data from the follow-up within one week after surgery and added a statement clarifying that hypotony did not persist. (Lines 116-118)

Moreover, the technique of intraocularpressure evaluation was not thoroughly described. This is essential for the study 's reproducibility.

Answer:

We added a description of how we measured IOP in the Methods section (Lines 72-74).

Reviewer #1:

-I would suggest to change "Our study clearly showed that suturing sclerotomy reduces the incidence of irreversible visual impairment due to severe hypotony in MIVS." (line 129) because the design is retrospective and not comparative, so this statement appears unbalanced.

Answer:

Thank you for your insightful comments. To avoid giving the impression of being overstated, we revised the wording from ‘reduced hypotony’ to ‘had a low incidence of hypotony’ and to ‘had no incidence of irreversible visual impairment due to severe hypotony’ in the Discussion section (Lines 165 and 220).

-I would also point out that there is a discrepancy in numbers: eyes with ERM (line 58: 144 eyes; line 101: 114 eyes), eyes with RRD (line 59, line 101)

Answer:

We corrected it (Lines 62 and 113).

-Then I noticed that the conclusion "Eyes with air or gas tamponade had a significantly 104 lower incidence of hypotony compared to those with no tamponade (p=0.0498)." appears to be an artifact. In fact, air has 13. 3% of hypotony, while no tamponade has 6.3%. Please change accordingly.

Answer:

Thank you very much for your comments. As you indicated, it was misleading and we changed to compare no tamponade group and SF6 group instead of comparing no tamponade group and the sum of SF6, C3F8 and air group. (Lines 29-31, 118-119, 219, 222 and table1)

-Please specify how long it was the follow up in "Re-detachment in RRD eyes 5 (4,4%)"

Answer:

We included the duration of follow-up (Lines 153-155).

-Insufficient statistical analysis: PLOS requires reporting of effect estimates with 95% confidence intervals, not p-values alone.

Answer:

We added 95% confidence intervals (table 1 and Lines 118-130).

-Please perform a multivariable logistic regression for postoperative hypotony, including as covariates: reoperation status, type of tamponade (none/air/SF6/C3F8), surgical indication, combined phacoemulsification (yes/no), operative time, preoperative IOP, age, and sex.

Answer:

Thank you for your helpful comments. We performed a multivariable logistic regression analysis (Lines 96-98, 127-130 and Table 3). Surgical indication was excluded from the analysis due to the large number of categories and the small sample size within many of them, which could compromise model stability.

-I noticed that there are 458 eyes and 438 patients: please verify if a inter-eye bias does exist. I suggest a mixed model to address this potential bias.

Answer:

Thank you for your suggestion. We performed mixed-effects models (Table1), and added this in the Methods section (Lines 89-91).

-Misspelling: "guage" --> gauge "retionoschisis"--> "retinoschisis"

Answer:

We corrected them (Table 1 and Table2).

Reviewer #2:

1. The absence of a comparative control group (e.g., cases without suturing of sclerotomies) makes it difficult to determine the true impact of universal suturing on the rate of postoperative hypotony. Without direct comparison, the claim that suturing reduces hypotony remains speculative.

Answer:

Thank you very much for your insightful observation. We revised the wording from ‘reduced hypotony’ to ‘had a low incidence of hypotony’ and to ‘had no incidence of irreversible visual impairment due to severe hypotony’ to avoid overstatement (Lines 165 and 220). For ethical reasons, we could not establish a sutureless group, as previous reports have indicated that postoperative hypotony after sutureless vitrectomy is a well‑recognized complication. This limitation has been described in the Discussion section (Line 214).

2. The study evaluates intraocular pressure only on postoperative day 1. Since hypotony may develop or resolve over time, the lack of longitudinal follow-up data limits the ability to understand the persistence or clinical relevance of hypotony beyond the immediate postoperative period.

Answer:

We appreciate your concern. We included data from the follow-up within one week after surgery and added a statement clarifying that hypotony did not persist. (Lines 116-118)

3. The manuscript does not clearly specify the instrument and method used for intraocular pressure assessment. Given that small variations in measurement technique (e.g., Goldmann applanation vs. non-contact tonometry vs. rebound tonometry) can significantly influence IOP values, clarification is essential for the validity of the results.

Answer:

We expanded the description of how we measured the IOP in the Methods section (Lines 72-74).

4. The paper does not elaborate on whether conjunctival displacement or beveled versus straight insertion techniques were used. These factors are known to influence wound closure and postoperative leakage risk. Their omission makes it difficult to generalize the findings or compare them with prior literature.

Answer:

We described the techniques in the method section (Lines 82-83).

5. Being a retrospective review from one institution, the results may reflect local surgical techniques, case mix, or surgeon preference rather than generalizable outcomes.

Answer:

We concur with the reviewer that, as this is a retrospective review from a single institution, the results may reflect local surgical techniques, case mix, or surgeon preference rather than generalizable outcomes. We have already acknowledged this point as a limitation in the Discussion section (Lines 214-216).

6. The series included a wide variety of vitreoretinal pathologies (ERM, RRD, MH, VH, PDR, etc.), yet the outcomes were pooled together. Since different conditions may carry different risks of postoperative hypotony, this heterogeneity makes interpretation less clear.

Answer:

We agree that the inclusion of various vitreoretinal diseases represents a limitation of this study, and we have addressed this point in the Discussion section (Lines 214-215).

7. Hypotony was defined strictly as IOP < 5 mmHg on day 1. While this is a common definition, functional hypotony (associated with maculopathy or choroidal changes) or transient hypotony beyond day 1 is not captured.

Answer:

We added OCT findings and a statement that hypotony did not persist. (Lines 70-71 and 115-118).

8. The study focuses almost exclusively on IOP, but does not address visual acuity, refractive change, or patient-reported outcomes (such as suture-related discomfort), which are relevant when recommending routine suturing.

Answer:

We appreciate your insightful comment. In the Discussion section, we have already addressed discomfort from sutures, surgically induced astigmatism, and longer operating time (Lines 207-209). While our study primarily focuses on intraocular pressure, we agree that discomfort from sutures and refractive changes are important considerations. We clarified this in the Discussion to emphasize that physicians should weigh these factors together with the risk of postoperative hypotony when deciding whether to suture sclerotomies (Lines 212-213).

9. Given the lack of a control group, limited time point, and retrospective design, the conclusion that suturing "reduces irreversible visual impairment" is overstated and not directly supported by the presented evidence.

Answer:

Thank you very much for your invaluable comments. To prevent overstatement, we revised the conclusion (Lines 165 and 220).
